# Patient death in simulation-based medical education

**DOI:** 10.5116/ijme.55f2.7d9b

**Published:** 2015-09-15

**Authors:** Lukas P. Mileder, Christian Vajda, Thomas Wegscheider

**Affiliations:** 1 Division of Neonatology, Department of Paediatrics and Adolescent Medicine, Medical University of Graz, Graz, Austria; 2 Department of Medical Psychology and Psychotherapy, Medical University of Graz, Graz, Austria; 3 Department of Anaesthesiology and Intensive Care Medicine, Medical University of Graz, Graz, Austria

**Keywords:** simulation-based, medical education, patient death

## Introduction

*‘Deficit omne quod nascitur’*, the Roman rhetorician Marcus Fabius Quintilianus wrote in his opus magnus “Institutio oratoria”. As death is an immanent part of human life, death and the medical profession are inseparably connected. The process of dying and death is complex, spanning the continuum of palliative care, diagnosis and certification of death, performance of last offices, and support of relatives.^1^ Literature shows that a patient’s death may have a strong emotional impact on healthcare professionals irrespective of their level of expertise.^2^ However, evidence suggests formal undergraduate education and practical training in caring for dying patients and dealing with patient death to be highly variable and underrepresented.^3^

According to DeMaria and colleagues^4^, psychological fidelity in simulation-based medical education (SBME) is frequently neglected in favour of equipment fidelity. Yet, adding emotional stressors to SBME has been shown to result in superior practical competency during standardized assessments,^4^ representing another reason to include patient death as specific learning goal in SBME.

In our opinion the topic of patient death in SBME is of importance to medical educationalists. Therefore, the aim of this article is (i) to offer our experience with delivering simulation-based training in emergency medicine and acute care for undergraduate medical students and (ii) to briefly review and discuss relevant literature.

### Simulated patient death

We have developed and implemented an elective course aiming at engaging medical students in full-scale simulation training in a simulated emergency department.^5^ Scenarios cover emergency management of patients with allergic reactions and cardiac diseases, featuring acute emergencies, (semi-)elective admissions and in-patient complications. Employed simulation-based instructional methodologies include high-fidelity patient simulators, standardized patients and hybrid simulation. Prior to training in the simulated emergency department, students not only receive an introduction to principles of crisis resource management, but also learn and practice how to deal with dying patients and delivery of bad news with a specific focus on relatives. Furthermore, students are explicitly informed during pre-briefing about the possibility of simulated patient death.

As part of training in the simulated emergency department, three to four students act as one medical emergency team, each of which is confronted with the death of a single patient as specific learning objective. After evaluation and clinical care, students are asked to diagnose the patient’s death and to inform and support relatives. All scenarios are scripted in advance, ensuring the simulator’s death to occur irrespective of students’ actions. Specially trained tutors for psychosocial crisis intervention and coping mechanisms are made available during simulation training as a precautionary measure in case of acute stress reactions. Following training in the simulated emergency department, thorough debriefing is conducted in order to minimize potential distress on behalf of participating students.

The course has been held three times with a total of 79 students participating. Students’ reactions have been very positive so far. Based on semi-structured feedback following debriefing, students perceived scenarios involving patient death as challenging, but welcomed the chance to practice certain skills including delivery of bad news to relatives in a realistic, yet safe environment. Most students found nontechnical skills training to be helpful for scenario performance. Availability of psychological support was mentioned positively several times, although there has been no need for acute psychosocial crisis interventions following training since implementation of the elective course.

## Discussion

Dealing with dying patients and patient death should be integral aspects of healthcare education and training. SBME provides trainees with the chance to practice relevant skills related to patient death in a safe setting. According to our experience, we suggest to offer dedicated training focusing on relevant psychological aspects prior to SBME and to specifically design scenarios involving simulated death.

It has been argued that SBME involving patient death should be grounded in sound ethical principles and that simulated death should not be used as punishment.^6^^,^^7^ We could not agree more with these recommendations, as creating a safe, honest and trustful learning environment is a major prerequisite for effective delivery of SBME. However, as “death is a realistic consequence of action taken or not taken”,^8^ there may be instances of severe (active or passive) medical malpractice where the facilitator may want to let the simulator die unexpectedly. Leighton and colleagues^9^ defined this type of death scenario as being unexpected both by the facilitator and the learners. Based on our experience, we have come to the conclusion that simulator death should be allowed in such situations, on condition that they are professionally accompanied as described before. As patients would be expected to die following medical failure in the actual healthcare environment, this practice does not only improve psychological fidelity of SBME, but may also enhance learning.^10^ However, we suggest following predefined operating procedures in these instances, including balancing of emotions while focusing on the incident and delivery of a detailed, careful debriefing.^7^

### Conflicts of interest

The authors declare that they have no conflict of interest.
